# 60. Penicillin Allergy Delabeling Program in the Post-acute Care Setting

**DOI:** 10.1093/ofid/ofab466.262

**Published:** 2021-12-04

**Authors:** Joseph Galipean, Jerry Jacob

**Affiliations:** 1 Hospital of the University of Pennsylvania, Philadelphia, Pennsylvania; 2 University of Pennsylvania, Philadelphia, Pennsylvania

## Abstract

**Background:**

A significant proportion of inpatients labeled with penicillin allergies do not have a true IgE-mediated hypersensitivity, which may unnecessarily limit options for treatment of infection and lead to suboptimal antibiotic selection. Post-acute care settings may provide a unique opportunity to capture patients at risk for adverse outcomes related to penicillin allergy labels. The objective of the study was to assess the feasibility and impact of a penicillin delabeling program in an inpatient rehab setting.

**Methods:**

We conducted a prospective observational study. Inpatients with penicillin allergies were identified weekly by manual review of electronic medical records. A clinical pharmacist reviewed each patient’s chart and identified patients for inclusion. Patients were excluded if they had a history of IgE-mediated hypersensitivity to penicillin within last 5 years, a history of a non-IgE mediated hypersensitivity, were severely immunocompromised, or were prescribed a contraindicated medication.

**Results:**

A total of 72 charts were reviewed over nine months, and 37 (51.4%) had their penicillin allergy updated to reflect prior beta-lactam tolerance. Of the 72 patient that were evaluated, 28 (38.9%) were eligible for potential penicillin allergy delabeling, and 44 (61.1%) were ineligible. 59 (81.9%) of the patients had a moderate-high risk allergy, 12 (16.6%) had a low risk allergy, and 1 (1.4%) had an intolerance. Of the 28 eligible patients, 11 (39.3%) had their allergy removed, 13 (46.4%) deferred testing, and 4 (14.2%) could not be tested due to staffing. Of the 28 patients that had their allergy delabeled, 7 (21.4%) had their allergy removed by MAR review, 2 (7.2%) had a skin test with a negative result, and 2 (7.2%) had a direct oral challenge with a negative result.

**Conclusion:**

A penicillin allergy delabeling program using a collaborative physician-pharmacist team model efficiently removed reported allergies in post-acute care patients. The post-acute care setting is an opportune environment to conduct a penicillin allergy delabeling program for patients not currently needing acute medical treatment.

**Disclosures:**

**All Authors**: No reported disclosures

